# Intrathecal Drug Delivery as a Rescue Strategy in Patients with Spinal Cord Stimulation: A Single-Center Case Series

**DOI:** 10.3390/jcm15124518

**Published:** 2026-06-11

**Authors:** Nicolás Cordero-Tous, Marcos Salmerón-Martín, Bernardino Alcazar-Navarrete, Manuel Cortiñas-Sáez, Rafael Gálvez-Mateos, Manuel Alejandro Sánchez-García

**Affiliations:** 1 Functional Neurosurgery Unit, Department of Neurosurgery, Hospital Universitario Virgen de las Nieves, Juan Pablo II Avenue, S/N, 4th Floor, 18013 Granada, Spain; 2 Pain Unit, Department of Anesthesiology, Reanimation and Pain Management, Hospital Universitario Virgen de las Nieves, Juan Pablo II Avenue, S/N, 2nd Floor, 18013 Granada, Spain; marcos.sm9290@gmail.com (M.S.-M.); mcortinassaenz@gmail.com (M.C.-S.); rafaelgalvez@hotmail.com (R.G.-M.); 3 Department of Pulmonolgy, Hospital Universitario Virgen de las Nieves, Juan Pablo II Avenue, S/N, 2nd Floor, 18013 Granada, Spain; balcazarnavarrete@gmail.com; 4 Medicine Department, University of Granada, 18011 Granada, Spain

**Keywords:** spinal cord stimulation, spinal pain, chronic low back pain, intrathecal infusion, chronic non-cancer pain, health related quality of life

## Abstract

**Background:** Chronic pain is the leading cause of years lived with disability worldwide, with low back pain representing the most prevalent and disabling condition. Spinal cord stimulation (SCS) and intrathecal drug delivery (IDD) systems are established neuromodulation techniques for refractory chronic pain. However, a subset of patients experiences partial or declining benefit with either modality alone. In such cases, combined therapy may represent a rescue strategy. **Methods**: retrospective case series at a single center, including patients previously implanted with SCS who subsequently required IDD due to loss of efficacy or inadequate pain coverage. Pain intensity, opioid consumption, health-related quality of life, and patient satisfaction were assessed using validated instruments. **Results**: Twelve patients were included. Persistent low back pain with mixed nociceptive–neuropathic features was the most common indication. Combined therapy was observed in association with a mean reduction of 3.5 points on the Numeric Rating Scale, corresponding to an approximate 40% decrease in pain intensity. More than half of the patients discontinued systemic opioids. Complications occurred in seven patients (58.3%), mostly hardware-related and manageable with surgical revision; only one patient developed a device-related infection. **Conclusions**: In this case series, combined SCS and IDD therapy was observed in association with clinically meaningful pain reduction, decreased opioid use, and high patient-reported satisfaction. Although quality-of-life scores remained below population norms, patients consistently reported subjective improvement. Combined neuromodulation may represent a valid rescue option in selected patients with insufficient response to SCS alone.

## 1. Introduction

Chronic pain and related diseases are the leading cause of the number of years the population lives with disability [1], with low back pain being the most common and the leading cause of disability. Refractory chronic pain imposes significant physical and mental limitations and suffering on sufferers, affecting all aspects of their lives and leading to a quality of life that is worse than that of the general population [2,3]. Therefore, there is an urgent need to explore new alternatives for the treatment of chronic pain in these patients, especially those with refractory pain [4,5].

Spinal cord stimulation (SCS) is a neuromodulation technique that delivers electrical stimulation to the dorsal columns to modulate pain signaling, while intrathecal drug delivery (IDD) provides continuous administration of analgesic agents directly into the cerebrospinal fluid. Conventional SCS targets the dorsal columns of the spinal cord through epidurally placed leads, modulating ascending nociceptive transmission and descending inhibitory pathways. It has traditionally been used for widespread neuropathic pain syndromes. Intrathecal drug delivery systems, commonly referred to as intrathecal pumps, constitute an important salvage option in patients with refractory chronic pain who experience inadequate analgesia. These systems deliver analgesic agents directly into the cerebrospinal fluid, allowing significantly lower doses than systemic administration while achieving higher concentrations at spinal receptor targets and minimizing systemic adverse effects. Commonly employed agents include morphine, hydromorphone, ziconotide, and combination regimens tailored to the patient’s pain phenotype. Both therapies are included in the fourth tier of the WHO analgesic ladder and are indicated for refractory chronic pain conditions, including persistent spinal pain syndrome (formerly failed back surgery syndrome), neuropathic pain, and complex regional pain syndrome [6,7,8].

Despite proven efficacy, up to 30% of patients treated with SCS and approximately 10% treated with IDD experience inadequate or diminishing benefit over time. When SCS fails or loses efficacy over time, several rescue strategies may be considered before abandoning neuromodulation altogether. These include lead revision in cases of migration or suboptimal coverage, reprogramming with alternative waveforms (high-frequency stimulation, burst stimulation, closed-loop stimulation, or multipolar configurations), conversion from conventional tonic SCS to newer stimulation paradigms [9,10]. Combined SCS-IDDS therapy may therefore be considered not merely as escalation, but as a complementary multimodal rescue strategy for highly selected patients. In these cases, the combination of both modalities may act as a rescue therapy, defined as the addition of a second neuromodulation strategy following partial response or loss of efficacy of the initial intervention.

The aim of this study is to describe our single-center experience with combined SCS and IDD therapy in patients with refractory chronic pain who did not achieve satisfactory outcomes with SCS alone, supporting the limited evidence in the literature [11,12,13,14,15].

## 2. Materials and Methods

The design of the study was a retrospective observational case series conducted at a single center between September 2023 and March 2024. The study protocol was approved by the Granada Provincial Ethics Committee (CEI/CEIM code: 1666-N-23). All participants agreed to participate and signed an informed consent form.

### 2.1. Study Population

**Figure 1:**
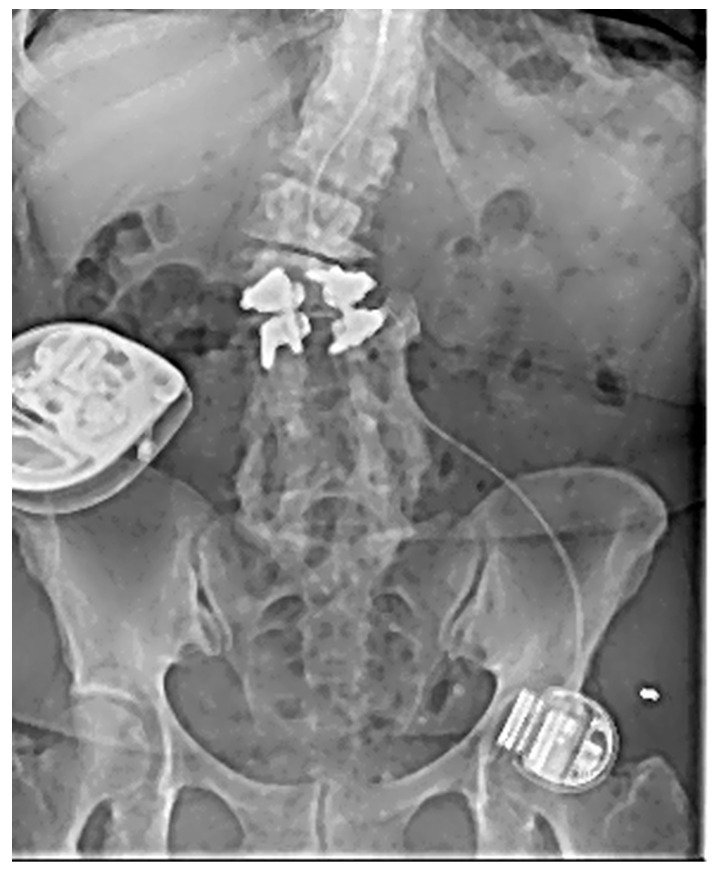
Example of an X-ray of a patient with combined therapy (SCS + IDD).

Adult patients with refractory chronic pain managed by the Pain Unit of Virgen de las Nieves University Hospital (Granada, Spain) who had previously undergone SCS implantation and subsequently received an IDD system (SynchroMed II^®^, Medtronic, Minneapolis, MN, USA) were included (Figure 1). The data were extracted from the medical records of treated patients and from scheduled consultation visits.

**Figure 2:**
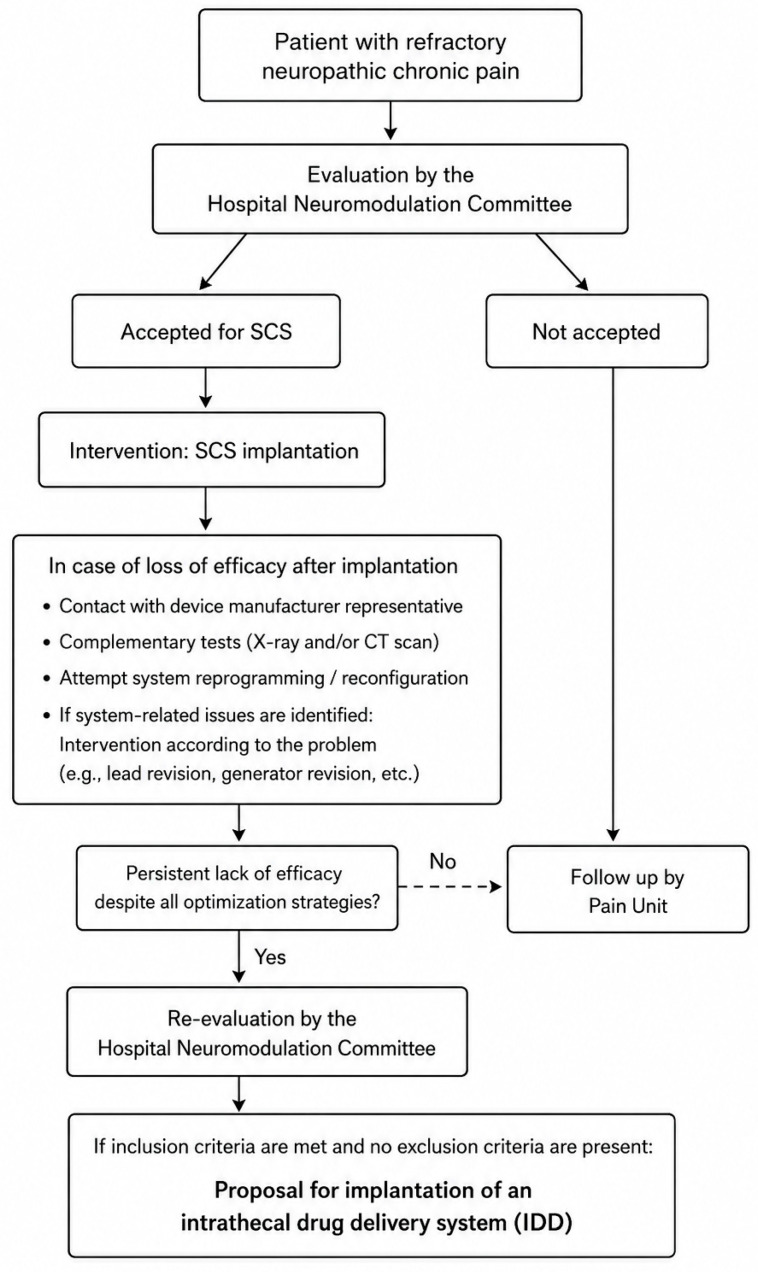
Flowchart for selecting rescue patients for failed posterior cord stimulation using intrathecal infusion pumps. Abbreviations: SCS, spinal cord stimulation.

Inclusion criteria were refractory chronic pain, treatment with an intrathecal analgesia device and a spinal cord stimulator for more than 3 months, being of legal age and consenting to participate in the study by signing the informed consent form. Patients who were unable to respond to the study questionnaires, patients who had received combined treatment for less than 3 months and patients who refused to participate were excluded. The study is being conducted thanks to the close collaboration between the anesthesiology department and the neurosurgery department. The SCS was implanted first in all patients, using paddle or percutaneous electrodes depending on the patient and the chronic pain committee’s decision. After a loss of efficacy was detected, or due to insufficient coverage of their lumbar pain, the intrathecal drug delivery system was implemented. The criteria established to determine whether a loss of system effectiveness had occurred, after excluding migration and technical system problems, were a progressive increase in parameters suggesting the development of perielectrode fibrosis, the appearance of bothersome dysesthesias, or the inability to achieve significant relief with program adjustments. In all cases, every possible measure was taken to optimize the system by both the medical team and the SCS-trained technical staff. The fact that a substantial number of patients were successfully salvaged following the established treatment algorithm limited the study cohort to those cases in which no other therapeutic alternatives remained (Figure 2).

### 2.2. Study Objectives

The primary objective of the study was to analyze the efficacy of combined spinal cord stimulation and intrathecal infusion therapy for the treatment of refractory chronic pain, assessing reduction in pain intensity. Secondary outcomes included opioid consumption, health-related quality of life, and patient satisfaction. Given the descriptive nature of the study, no a priori hypothesis testing was planned.

### 2.3. Variables

Baseline demographic and anthropometric data were collected from all participants, including age, sex, height, body mass index (BMI) and educational level. In addition, other clinical data were collected, such as the etiology of chronic pain, type of pain, number of years with each device, differences in analgesic use prior to implantation and current opioid use, dysthymia prior to implantation, and type of medication used in the infusion device. To assess differences in patients’ pain levels prior to device implantation and their current pain, the Numeric Rating Scale (NRS) was used through a review of patients’ medical records. Validated health questionnaires, including the SF-12 [16] (which analyzes the patient’s quality of life multidimensionally using 12 questions and divides it into mental [MCS-12] and physical [PCS-12] domains, with a mean of 50 for each score), were administered on the day of the examination to assess the patients’ health-related quality of life (HRQoL); the Brief Pain Inventory Short Form (BPI-SF) [17,18], which assesses patients’ impairment of quality of life due to pain using 11 questions ranging from 0 (no pain/no impairment) to 10 (most severe pain/most severe impairment), giving an overall score between 0 and 110 [17]; and the European Quality of Life-5 Dimensions (EQ-5D-5L) [19,20,21,22], which uses 5 questions on relevant aspects of the patient’s life (mobility, self-care, daily activities, pain/discomfort and anxiety/depression) with 5 levels of severity (no problems, mild problems, moderate problems, severe problems, severe problems, severe problems and incapacitated) relates each patient to their reference population and allows comparisons with patients with other pathologies based on their indexed score, which ranges from 0 (the lowest possible score corresponding to death) to 1 (the best possible health status). The Patient Global Impression of Improvement (PGI-I) scale was used to assess patients’ overall impression of the application, outcome and tolerability of the devices, ranging from 1 to 7, with 1 being the best possible rating (very much better) and 7 being the worst possible (very much worse). In addition, a health survey was conducted on the day of the consultation (visual analog scale of the EQ-5D questionnaire [EQ-5D VAS]), which ranged from 0 (worst possible health status) to 100 (best possible health status) [23].

### 2.4. Statistical Analysis

Data are presented using measures of central tendency and dispersion (mean and SD) for continuous variables and as a number (and percentage of total) for ordinal variables. Comparison of normally distributed continuous variables was performed using Student’s *t*-test (NRS improvement). The association between variables was assessed using Spearman’s rank correlation coefficient (Spearman’s ρ), a non-parametric measure that evaluates the strength and direction of a monotonic relationship between two variables based on ranked data, without assuming normality. This approach was adopted given the limited sample size in an effort to minimize potential sources of bias as much as possible. Values of ρ range from −1 to +1, where ρ = +1 indicates a perfect positive monotonic association, ρ = −1 indicates a perfect negative monotonic association, and ρ = 0 indicates the absence of a monotonic relationship. All statistical analyzes were performed using the Jamovi statistical package version 2.6 (The Jamovi Project, 2024, Sydney, Australia). A *p*-value < 0.05 was considered statistically significant. Given the small sample size, inferential statistics were interpreted cautiously and primarily used for exploratory purposes.

## 3. Results

A total of 12 patients were included in the study (4 women and 8 men). The age ranged from 42 to 72 years (mean age 55 years). An overview of our study population can be found in Table 1.

The main indication for implantation of both devices was persistent low back pain, with mixed pain being the most common (pain with nociceptive and neuropathic features). The SCS were placed at the thoracic epidural level. In addition, all patients with IDD were treated with morphine as drug, and the catheter was placed at the thoracic level in the intrathecal space. A significant decrease in opioid consumption was observed in patients treated with both devices. More than half of the patients were not taking opioids at the time of the study, and 25% of them were treated with weak opioids. The specific clinical and technical characteristics of the participants are listed in Table 2 and Table 3.

**Figure 3:**
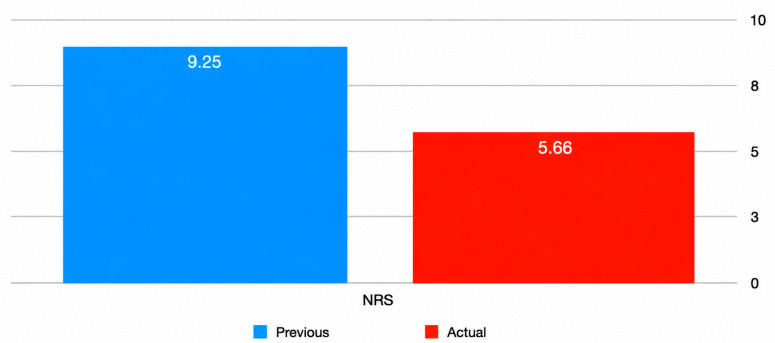
The figure shows the initial NRS score (Blue) and the decrease that occurs after the application of the intrathecal pump system (red). *p* < 0.001. Abbreviations: NRS; numeric rating scale.

When examining the NRS scores (Figure 3), we found a clinically meaningful reduction in pain compared to baseline (approximately 40%), with a 3.5-point decrease in pain compared to the initial assessment (*p* < 0.001). When analysing the quality of life data (Table 4), we found that the numerical values were below the mean.

**Figure 4:**
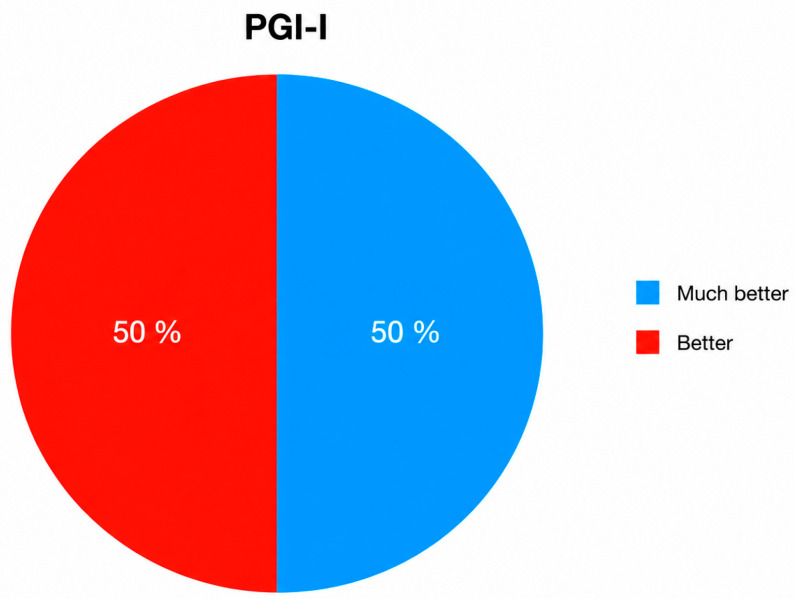
Patient Global Impression of Improvement scale (PGI-I). *p* < 0.001.

When analysing patient satisfaction with the treatment using the PGI-I survey, we found that the majority reported feeling “better” or “much better” as a result of the treatment (Figure 4). Dysthymia was observed in 66% of the study population at the start of treatment.

As far as complications are concerned, only one patient had a system infection (8.3%). In terms of SCS therapy, two patients underwent electrode repositioning to optimize efficacy, and only one presented with painful pocket symptoms, which were treated conservatively. In all cases, only one additional procedure was required. For IDD, one patient required multiple procedures due to a systemic infection. (Table 2).

**Figure 5:**
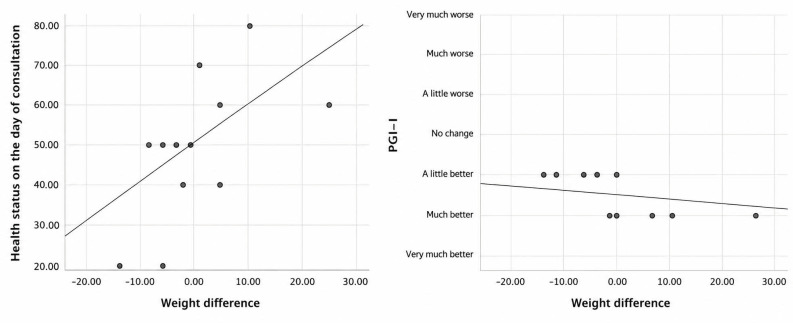
Simple scatter plot with fitted line of Health status on the day of consultation by Weight difference, *p* < 0.025 (**left**) and simple scatter plot with fitted line of PGI-I by Weight difference, *p* < 0.002 (**right**). Abbreviations: PGI-I; Patient Global Impression of Improvement scale.

**Figure 6:**
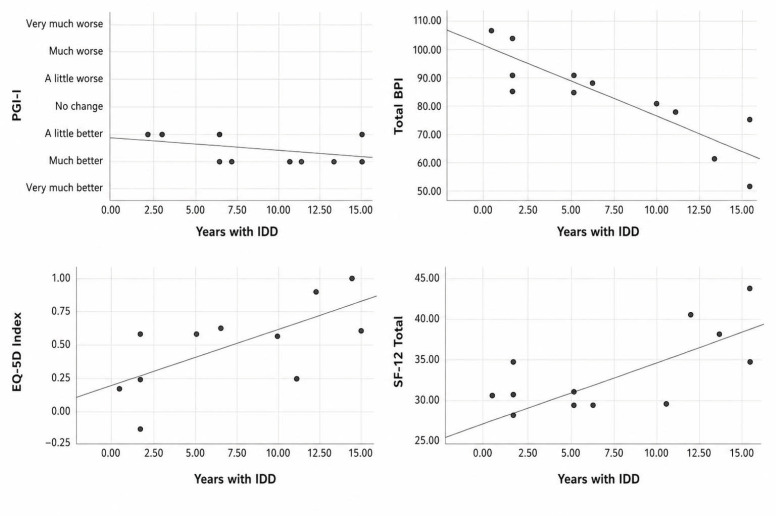
Simple scatter plot with fitted line of Years with IDD by PGI-I scale (*p* < 0.045), BPI (*p* < 0.001), SF-12 total (*p* < 0.044), and EQ-5 index (*p* < 0.007). Abbreviations: IDD; intrathecal drug delivery, PGI-I; Patient Global Impression of Improvement scale, BPI; Brief Pain Inventory, EQ-5D; European Quality of Life-5 Dimensions.

Statistical analysis using Spearman’s rank correlation coefficient, comparing demographic variables with quality of life and patient satisfaction measures, revealed significant relationships in two cases. First, a relationship was observed between weight gain and health status on the day of the consultation (r = 0.640, *p* < 0.025) and the PGI-I (r = −0.798, *p* < 0.002) (Figure 5). Second, a relationship was found between the duration of treatment with IDD and improvements in the quality of life scales: BPI (r = −0.908, *p* < 0.000), SF-12 total (r = 0.588, *p* < 0.044), and EQ-5 index (r = 0.073, *p* < 0.007). This relationship was also observed with improvement in the PGI-I scale (r = −0.586, *p* < 0.045) (Figure 6).

## 4. Discussion

IDD and SCS are widely established and have led to positive outcomes in both pain management and quality of life [24,25,26,27,28]. Traditionally, SCS has been used to treat neuropathic pain, such as persistent low back pain with accompanying radiculopathy, complex regional pain syndromes (in type I, but more frequently in type II, which show nerve damage) and phantom limb pain. In recent years, several therapeutic targets of different types have emerged, including movement disorders and diabetic neuropathy [29,30]. However, the indication for IDD is closely linked to the type of drug used. Morphine and ziconotide are mainly used for the treatment of chronic pain [8,24,31,32]. However, the combined use of both therapies (SCS + IDD) is uncommon and has only been described in a few cases in the literature, with use in refractory chronic pain being even rarer [11,13,14,15]. There are not many centers where both techniques are used, and our center is one of them.

The authors present one of the longest series published to date looking at the salvage of patients with partially effective SCS therapy supplemented with IDD to control pain and improve the patient’s quality of life. Traditionally, SCS therapy has provided about 50 pain relief in about 50% of patients [33]. These figures have improved over the years with advances in technology, particularly with the introduction of new waveforms, reaching almost 90% in clinical trials and fluctuating between 60–70% in routine daily practice [34,35]. Of particular concern has always been the situation of patients in whom SCS therapy has failed, even if their proportion is relatively low. For this reason, the rescue of patients who have received this therapy but whose efficacy has diminished is not unknown in the literature [36,37].

The reasons for rescue therapy can be manifold. The main reason has traditionally been that SCS therapy was more effective for pain radiating to the extremities than for axial pain. Although this problem has been partially resolved with the advent of new waveforms [36], it still occurs. Other reasons include loss of efficacy due to fibrosis, failure of system components, numerous complications, and the occurrence of other pain conditions. The usual method of correcting these events has been to re-implant the SCS system to regain adequate pain coverage [37]. More recently, attempts have been made to replace low-frequency stimulation with systems that are compatible with new waveforms. Good results have been obtained, ranging from 62% to 83% improvement [36,37]. However, the use of IDD as a rescue therapy for SCS is not widespread. In our case, we used IDD primarily as an adjunct to SCS therapy to provide relief in areas that could not be adequately addressed by SCS, thereby improving not only pain control but also quality of life.

Overall, the combined use of both therapies has only been described in a few scientific articles. The first to report it was Lind et al. [11] in a study that attempted to rescue 48 patients in whom SCS therapy was ineffective. Among the various options they used, 7 patients received intrathecal baclofen concomitantly with SCS therapy, resulting in an improvement in VAS from 8 to 3; however, 2 of them later had to be explanted due to complications. In another 4 cases, IDD was used as monotherapy after removal of the SCS. Later, Tomycz et al. [14] presented a series of 11 patients with persistent lumbar pain in whom SCS and IDD systems were not installed simultaneously. Initially, 8 patients had an SCS and 3 had an IDD, and the other therapy was added. They reported that all patients experienced an improvement in pain, with 55% considering the IDD therapy to be superior, while quality of life improved in 82% of cases with the combined therapy. Schechtmann et al. [13] suggested the administration of intrathecal injections in patients with SCS who had experienced a loss of efficacy or whose study phase had not been favorable. Although they reported that 7 out of 10 patients improved with the intrathecal trial of baclofen and clonidine, only 2 patients were ultimately implanted with an IDD system, as the others experienced adverse drug effects. The improvement in these two patients was between 32% and 82%, depending on the medication used. In a more recent series, Staudt et al. [15] performed a retrospective analysis of their cohort of 11 patients with SCS (10 patients) or IDD (1 patient) who had experienced a loss of efficacy and were implanted with the other system in combination. The authors analyzed the differences between baseline and the second intervention, both in terms of pain control and quality of life. They observed a statistically significant improvement in pain intensity as measured by a decrease in NRS from 7.64 to 4.82.

In our series, we analyzed 12 patients with SCS therapy that was only partially effective (loss of efficacy after previous good efficacy or incomplete coverage) and was complemented by IDD therapy with morphine as drug. It is important to note that patients are able to differentiate the effect of each system and that they are dependent on both, so that when the battery of one of the systems is depleted, their pain reappears partially or in certain areas. Therefore, we hypothesize that the combination of both systems may contribute to improved outcomes that the combination of both systems improves their condition. With the combined therapy, the improvement in pain control was of 3.5 points on the NRS observed (associating the previous improvement obtained with the SCS with the current improvement at other levels with IDD). In terms of quality of life, scores were below average, and on the Patient Global Impression of Improvement (PGI-I) scale, all patients rated their satisfaction as level 2 or 3 (better or much better). These data are similar to previous publications suggesting that the use of combination therapy as a rescue option leads to favorable outcomes. Noteworthy is the reduction in opioid consumption among these patients, leading to decreased physical and psychological adverse effects associated with opioid use, consequently improving the HRQoL. These findings suggest a HRQoL improvement with combined therapy from both physical and mental perspectives, despite scores still being lower than those of the reference population, associated with pain reduction, reduction in opioids consumption and positive treatment satisfaction.

Our study also demonstrates a progressive improvement in patients’ quality of life and treatment satisfaction over time with combined SCS and IDD therapy, showing a statistically significant linear correlation. To our knowledge, this temporal association has not been previously reported in the literature. This finding may be explained by gradual patient adaptation to their situation, together with the optimization of intrathecal drug dosing and stimulation parameters, which typically occurs over extended follow-up, as suggested by previous studies in patients with IDD [28].

Due to the limited number of centers performing SCS and IDD and the potential cost, the use of combination therapy may not be widespread. However, it is important to emphasize that we believe that this combined therapy is a cost-effective treatment that improves patients’ quality of life and relieves their pain. However, it is true that no cost-effectiveness studies have been conducted on this topic.

Possible complications resulting from SCS or IDD treatment are common. Rates of around 50% are reported in the literature [38]. In the case of SCS therapy, complications are often related to infections or electrode displacement [27]. In the case of IDD, infections and problems related to the intrathecal catheter are also common [38]. The combined use of both systems obviously increases the risk of complications with both systems. For example, Lind et al. [11] reported an explantation rate due to complications of 28% in their cases rescued with IDD. Both Tomycz et al. [14] and Staudt et al. [15] reported a very high complication rate, with 64% of patients requiring additional surgery. Schechtmann et al. [13] reported adverse effects of the drugs, but in terms of complications of the implanted systems, infections occurred in 2 out of 4 patients (50%) that did not require intervention. In our case, the complication rate requiring intervention for the IDD was very low, with most complications related to a recurrent infection. However, the complication rate for the SCS system increased to 25%. Nevertheless, most patients required only one procedure and only one patient required multiple surgeries. Importantly, no permanent neurological deficits were observed, and most complications were resolved with a single intervention. Overall, the likelihood of complications increases when both systems are used for a prolonged period of time (which includes changes to the pump or generator), a finding that is consistent with the literature and confirmed by our results.

### 4.1. Future Perspective

Future research should focus on defining the precise role of combined spinal cord stimulation (SCS) and intrathecal drug delivery (IDD) within the treatment algorithm for refractory chronic pain. Although our findings and previous reports suggest that this strategy may be an effective salvage option in selected patients with partial response or loss of efficacy after SCS, larger prospective multicenter studies are needed to identify predictors of response and establish standardized selection criteria [11,14,15]. The rapid evolution of neuromodulation technologies, including high-frequency SCS, burst stimulation, closed-loop systems, and dorsal root ganglion stimulation, may alter the timing and indication for IDD as a rescue strategy, requiring comparative studies against these newer approaches [34,35,36,37]. Similarly, optimization of intrathecal pharmacotherapy remains an important area of investigation, as alternative agents such as ziconotide or combination regimens may provide improved efficacy or tolerability compared with morphine alone [8,31]. Given the significant healthcare burden associated with refractory chronic pain, formal cost-effectiveness analyses are also warranted, particularly since dual-device therapy may potentially reduce long-term opioid consumption, improve function, and decrease healthcare utilization despite higher initial procedural costs. Technological improvements aimed at reducing complication rates, including device miniaturization, infection prevention strategies, and remote monitoring, may further enhance long-term safety and durability. Finally, future studies should prioritize patient-centered outcomes beyond pain intensity, including functional recovery, opioid independence, psychological well-being, and long-term health-related quality of life, as these measures may better reflect the true clinical value of combined neuromodulation strategies. Within this context, combined SCS and IDD therapy may evolve from a purely salvage intervention toward a more personalized therapeutic option in carefully selected patients with complex refractory pain.

### 4.2. Limitations

Our study has important limitations. The most important is the small sample size, which, although one of the largest in the current literature, is still insufficient to draw conclusive conclusions. Lack of sample size calculation. Single-centre bias. Potential selection bias (complex patients). In addition, the retrospective nature of the study requires cautious interpretation of the data. Furthermore, the study was conducted under routine clinical conditions, which provides an insight into real-life treatment. However, the large time span implies that different professionals were involved, and the criteria may not have been consistent in all cases. Nevertheless, despite these limitations, the authors believe that the study may help to improve the treatment of patients with chronic pain who have an SCS system that has become less effective for one reason or another.

## 5. Conclusions

Refractory chronic pain imposes significant limitations and suffering on patients, impacting them both physically and psychologically and affecting all areas of life. Overall, patients who received combined therapy (SCS and IDD) experienced pain relief, with most reporting that they felt better or much better after implantation of both devices. These results indicate an improvement in health-related quality of life with the combined treatment, both physically and psychologically, although it remains lower than that of the reference population. This improvement is related to a reduction in pain and positive satisfaction with the treatment, and our data also suggest that the longer the therapy is maintained, the better the results. These findings emphasize that combined therapy may be a valid option for patients for whom SCS treatment alone does not achieve satisfactory results; however, conducting studies with a larger patient cohort would be advisable to confirm the validity and reproducibility of these findings.

## Figures and Tables

**Table 1:** General overview of study population. All the data are expressed in mean (±SD) or n (% of entire column).

Variables	Global (n = 12)
Age.	55.4 ± 8.3
Height, cm.	169.9 ± 8.1
BMI previous to IDD.	29.3 ± 3.5
Years with IDD.	8.8 ± 4.8
Years between SCS and DIT.	3.5 ± 1.5
Age at IDD.	46.5 ± 8.1
Gender, male.	8 (66.7%)
Educational level.	
Compulsory Schooling or lower.	6 (50.0%)
Vocational training.	3 (25.0%)
High School or University Education.	3 (25.0%)
Diagnosis	
Persistent Low Back Pain.	10 (83.3%)
Complex Regional Pain Syndrome.	1 (8.3%)
Generalized Pain.	1 (8.3%)
Type of Pain.	
Somatic Nociceptive.	1 (8.3%)
Neuropathic.	2 (16.7%)
Mixed (Nociceptive/Neuropathic).	9 (75.0%)
Pain distribution.	
Right Lumbosciatica.	3 (25.0%)
Left Lumbosciatica.	8 (66.7%)
Complex Regional Pain Syndrome Type II in left foot.	1 (8.3%)
Performance of interventional techniques prior to implantation.	10 (83.3%)
Analgesic consumption prior to implantation.	
Non-opioid analgesia.	0 (0.0%)
Strong opioids.	12 (100.0%)
Current opioid consumption.	
No.	7 (58.3%)
Weak opioids.	3 (25.0%)
Strong opioids.	2 (16.7%)
Pre-implant Dysthymia.	8 (66.7%)
Intrathecal drug.	
Morphine.	12 (100.0%)
Ziconotide.	0 (0.0%)
Intrathecal morphine dose.	3.7 ± 2.1
Numeric Rating Scale (NRS).	
Previous.	9.3 ± 0.7
Actual.	5.6 ± 1.3

Abbreviations: IDD: Intrathecal Drug delivery. SD: Standard Deviation. BMI: Body Mass Index. SCS: Spinal Cord Stimulation.

**Table 2:** Clinical features of study population.

Patient	Age (y)/ Gender	Diagnosis	Pain Distribution.	Years from SCS to IDD	Years with IDD + SCS	Complications
1	53/F	PSPS	Right Lumbosciatica	3	7	No
2	52/M	Generalized pain	Right Lumbosciatica	5	8	No
3	47/F	PSPS	Left Lumbosciatica	2	3	No
4	57/M	PSPS	Left Lumbosciatica	7	12	No
5	60/M	PSPS	Left Lumbosciatica	4	14	No
6	72/M	PSPS	Left Lumbosciatica	4	11	No
7	50/F	PSPS	Right Lumbosciatica	4	16	Si
8	42/M	PSPS	Left Lumbosciatica	1	7	No
9	63/M	PSPS	Left Lumbosciatica	3	16	No
10	48/M	PSPS	Left Lumbosciatica	2	4	No
11	61/F	PSPS	Left Lumbosciatica	4	4	No
12	60/M	CPRS II	Left foot	3	4	No

Abbreviations: F; Female. M; Male. PLBP; Persistent low back pain. CRPS; Complex Regional Pain Syndrome. SCS; Spinal Cord Stimulation. IDD; Intrathecal Drug Delivery.

**Table 3:** Technical features of study population.

Patient	Electrode Type	Location	Waveforms	System	Reason for Failure of SCS	Intrathecal Morphine (Dose)
1	Surgical	Thoracic	BURST	ANS-StJude	Loss of efficacy	5.00 mg/day
2	Surgical	Thoracic	Tonic	ANS-StJude	Incomplete coverage	4.75 mg/day
3	Percutaneous	Thoracic	Tonic	Medtronic	Incomplete coverage	3.80 mg/day
4	Surgical	Thoracic	Tonic	ANS-StJude	Incomplete coverage	8.60 mg/day
5	Surgical	Thoracic	Tonic	Medtronic	Loss of efficacy	4.80 mg/day
6	Surgical	Thoracic	Tonic	Medtronic	Loss of efficacy	2.00 mg/day
7	Surgical	Thoracic	Tonic	Medtronic	Loss of efficacy	3.50 mg/day
8	Percutaneous	Thoracic	Tonic	Medtronic	Incomplete coverage	2.35 mg/day
9	Surgical.	Thoracic	Tonic	Medtronic	Incomplete coverage	0.70 mg/day
10	Percutaneous	Thoracic	Tonic	Medtronic	Incomplete coverage	1.40 mg/day
11	Percutaneous	Thoracic	HF10k	Boston	Incomplete coverage	3.40 mg/day
12	Percutaneous	Thoracic	Tonic	Medtronic	Incomplete coverage	4.30 mg/day

Abbreviations: HF10k; High Frequency 10.000 Hz.

**Table 4:** Health-related Quality of life of patients receiving combined Therapy with SCS + IDD.

HRQoL Scale	Value (Mean ± SD)
EQ5D5L index (values between 0–1)	0.5 ± 0.3
EQ5D VAS (values between 0–100)	49.1 ± 17.8
SF-12 (values between 0–100)	31.2 ± 5.5
SF-12—MCS-12 (values between 0–100)	34.7 ± 18.6
SF-12—PCS-12 (values between 0–100)	31.7 ± 22.6
BPI-SF (values between 0–110)	83.8 ± 15.8

Abbreviations: HRQoL: Health-related Quality of life. SCS: Spinal Cord Stimulation. IDD: Intrathecal Drug Delivery.

## Data Availability

The datasets presented in this article are not readily available because of ethical reasons.

## Abbreviations

The following abbreviations are used in this manuscript:

SCS	Spinal cord stimulation
IDD	Intrathecal drug delivery
NRS	Numeric Rating Scale
BMI	Body Mass Index
SF-12	Short Form 12 (mental [MCS-12] and physical [PCS-12])
HRQoL	Health-Related Quality of Life
BPI-SF	Brief Pain Inventory Short Form
EQ-5D-5L	European Quality of Life-5 Dimensions
PGI-I	Patient Global Impression of Improvement
EQ-5D VAS	Visual Analogic Scale of the EQ-5D questionnaire
PLBP	Persistent Low Back Pain
CRPS	Complex Regional Pain Syndrome
